# Selection of the Optimal Candidate to MitraClip for Secondary Mitral Regurgitation: Beyond Mitral Valve Morphology

**DOI:** 10.3389/fcvm.2021.585415

**Published:** 2021-02-03

**Authors:** Tanya Salvatore, Fabrizio Ricci, George D. Dangas, Bushra S. Rana, Laura Ceriello, Luca Testa, Mohammed Y. Khanji, Anna Laura Caterino, Corrado Fiore, Antonio Popolo Rubbio, Marianna Appignani, Maria Di Fulvio, Francesco Bedogni, Sabina Gallina, Marco Zimarino

**Affiliations:** ^1^Institute of Cardiology, “G. d'Annunzio” University of Chieti-Pescara, Chieti, Italy; ^2^Department of Cardiology, IRCCS Pol. S. Donato, S. Donato Milanese, Milan, Italy; ^3^Department of Clinical Sciences, Clinical Research Center, Lund University, Malmö, Sweden; ^4^Department of Neuroscience, Imaging and Clinical Sciences, “G. d'Annunzio” University of Chieti-Pescara, Chieti, Italy; ^5^Casa di Cura Villa Serena, Città Sant'Angelo, Pescara, Italy; ^6^Icahn School of Medicine at Mount Sinai, The Zena and Michael A. Wiener Cardiovascular Institute, New York, NY, United States; ^7^Imperial College Healthcare Trust, Hammersmith and Charing Cross Hospitals, London, United Kingdom; ^8^NIHR Barts Biomedical Research Centre, William Harvey Research Institute, Queen Mary University of London, London, United Kingdom; ^9^Barts Heart Centre, St. Bartholomew's Hospital, Barts Health NHS Trust, London, United Kingdom; ^10^Città di Lecce Hospital, Lecce, Italy; ^11^Interventional Cath Lab, Chieti, Italy

**Keywords:** MitraClip, heart failure, mitral regurgitation, cardiac magnetic resonance, echocardiography, multimodality cardiac imaging, mitral valve repair

## Abstract

Secondary mitral regurgitation (MR) occurs despite structurally normal valve apparatus due to an underlying disease of the myocardium leading to disruption of the balance between tethering and closing forces with ensuing failure of leaflet coaptation. In patients with heart failure (HF) and left ventricular dysfunction, secondary MR is independently associated with poor outcome, yet prognostic benefits related to the correction of MR have remained elusive. Surgery is not recommended for the correction of secondary MR outside coronary artery bypass grafting. Percutaneous mitral valve repair (PMVR) with MitraClip implantation has recently evolved as a new transcatheter treatment option of inoperable or high-risk patients with severe MR, with promising results supporting the extension of guideline recommendations. MitraClip is highly effective in reducing secondary MR in HF patients. However, the derived clinical benefit is still controversial as two randomized trials directly comparing PMVR vs. optimal medical therapy in severe secondary MR yielded virtually opposite conclusions. We reviewed current evidence to identify predictors of PMVR-related outcomes in secondary MR useful to improve the timing and the selection of patients who would derive maximal benefit from MitraClip intervention. Beyond mitral valve anatomy, optimal candidate selection should rely on a comprehensive diagnostic workup and a fine-tuned risk stratification process aimed at (i) recognizing the substantial heterogeneity of secondary MR and its complex interaction with the myocardium, (ii) foreseeing hemodynamic consequences of PMVR, (iii) anticipating futility and (iv) improving symptoms, quality of life and overall survival.

## Pathophysiology of Secondary Mitral Regurgitation

Mitral regurgitation (MR) can be classified as primary, because of a structural pathology of any component of the mitral valve (MV) apparatus, or secondary, when it occurs despite the MV being structurally normal, as a consequence of the underlying disease of the atrium or ventricle associated with various grades of annular dilation and/or distortion (1).

Compared with primary MR, secondary MR is more frequent and associated with higher mortality and risk of re-hospitalization in community studies, regardless of age, sex, and left ventricular (LV) ejection fraction (LVEF) (2).

In the context of ischemic or dilated cardiomyopathy, the primary determinant of secondary MR has been attributed to LV remodeling and dysfunction, promoting progressive imbalance between tethering and closing forces, eventually affecting mitral coaptation reserve. Chronic tethering promotes MV adaptation, retaining leaflet coaptation as long as compensatory valve growth is not exceeded by profibrotic processes, ultimately impairing closure and augmenting MR (3, 4). LV dyssynchrony is an additional co-determinant of secondary MR associated with adverse LV remodeling, systolic function impairment, and papillary muscle dysfunction, further hampering adequate leaflet coaptation and apposition (5, 6).

Secondary MR reflects a disease of the ventricle or atrium and can be classified as ischemic or non-ischemic MR, while atrial functional MR has only more recently been recognized in the literature (7).

In ischemic MR, the ischemic or infarcted myocardium is responsible for regional wall motion abnormalities, resulting in two distinct patterns of leaflet tethering, symmetrical, and asymmetrical. Symmetrical leaflet tethering pattern is characterized by the apical displacement of the subvalvular apparatus, with a symmetrical coaptation and a centrally directed jet of MR. The asymmetrical tethering pattern describes the predominant posterior displacement of the MV subvalvular apparatus, resulting in an eccentric posteriorly directed MR jet (Carpentier type IIIb) (8).

Non-ischemic secondary MR is the result of an underlying myopathic process characterized by globally impaired, progressively more dilated, and spherical LV causing displacement of the mitral subvalvular apparatus and annular dilatation (Carpentier type I). A predominant feature is the loss of normal mitral annular function, symmetrical leaflet malcoaptation and a centrally directed jet of MR (9). Compensatory mechanisms, including an adaptive increase in MV leaflet area, can preserve leaflet coaptation, but disease progression eventually leads to increasing MR severity.

Severe left atrial enlargement, most often seen with persistent or permanent atrial fibrillation, can lead to atrial functional MR. Isolated dilation and flattening of the mitral annulus from long-standing atrial fibrillation and atrial remodeling seems to play an important pathophysiological role (Carpentier type I) (7).

## Diagnostic Assessment and Prognostic Relevance

Secondary MR is independently associated with reduced survival and recurrent hospitalization in both patients with ischemic and non-ischemic cardiomyopathy (10), indicating that secondary MR should not be considered just a mere consequence of LV dysfunction, but a significant predictor of survival (1).

MR portends reduced life expectancy despite optimal medical therapy (OMT), with a mortality risk directly related to the effective regurgitant orifice area (EROA), peaking at 50% at 5 years for patients with EROA>40 mm^2^ (11). Quantitative thresholding for grading of secondary MR severity is a debated issue, as American and European guidelines endorsed different cut-offs (12, 13). The European Society of Cardiology (ESC) revised the American Heart Association/American College of Cardiology (AHA/ACC) definition of severe secondary MR by lowering EROA and regurgitant volume (RVol) cut-offs from 0.4 to 0.2 cm^2^ and from 60 to 30 mL, respectively (13), also highlighting the intrinsic limitations and lack of precision of 2D echo structural and Doppler parameters. Beyond EROA and RVol, regurgitant fraction (RF)—the ratio of RVol divided by stroke volume through the regurgitant valve—accurately reflects the hemodynamic severity of the regurgitant lesion and RF ≥50% has been proposed as an effective risk-based threshold significantly improving 5-year mortality discrimination compared with currently established algorithms (14).

Beyond 2D echocardiography, advanced applications of echocardiography—including 3D, strain imaging and particle imaging velocimetry (Echo-PIV)—and cardiovascular magnetic resonance (CMR) are considered key components of optimal patient selection and procedural planning.

3D echocardiography is extremely useful for grading of MR severity and to elucidate the underpinnings of secondary MR. The “en-face” anatomic visualization of the MV from both atrial and ventricular perspectives does provide unique information on the mechanisms of regurgitation, such as scallop prolapse, chordal rupture, clefts or restricted motion of leaflets; in addition, multiplane reconstruction enables axial imaging yielding a set of useful parameters for the selection of optimal candidate, including MV area, tenting height, tenting area, coaptive length, coaptive gap, interpapillary distances, anterior leaflet closing angle, posterior leaflet closing angle, and anterior leaflet inversion angle, with highly reproducible results (15, 16). The independence of 3D imaging on geometric assumptions further allows accurate quantification of LV dimensions and systolic function. This is particularly useful to establish appropriate timing of intervention, especially when CMR imaging is not readily available (17). Beyond morphological evaluation of MV, 3D color Doppler imaging provides a more detailed quantification of MR through direct assessment of vena contracta area, avoiding geometric assumptions and inaccuracies of the 2D PISA method (18, 19).

Strain imaging provides unique insight into complex cardiac mechanics and enables more precise evaluation of cardiac function. It has been shown to have potential clinical utility across the entire spectrum of valvular heart disease, including secondary. MR. Notably, LV global longitudinal strain (GLS) by speckle tracking echocardiography has been shown to be more sensitive to detect myocardial fibrosis than LVEF (20) and LV GLS <7% was independently associated with increased mortality in patients with significant secondary MR, whereas LVEF was not (21). Pending further evidence, deformation imaging abnormalities might play a role for risk stratification in asymptomatic patients with chronic secondary MR and better predict the optimal timing of valvular intervention.

EchoPIV is a velocity field imaging technique that applies optical analysis algorithms to sequential contrast-enhanced ultrasound images. Recently, an EchoPIV *in vivo* study on the acute effects of PMVR in patients with dilated cardiomyopathy, reduced LVEF and severe secondary MR, showed that MitraClip implantation has a significant impact on intraventricular flow dynamics yielding potentially harmful changes such as increase in energy dissipation and in flow force angle which are associated with negative LV remodeling (22). However, the possible link between these maladaptive changes and the suboptimal PMVR results reported in the literature (23) should be investigated in longitudinal studies.

CMR has been shown to offer a useful adjunct to echocardiography for the assessment of secondary MR by providing prognostically relevant clinical features including precise volumetric and functional measurements, myocardial scar or fibrosis assessment, and accurate quantification of mitral RVol and RF (24–26). In a prospective multicenter study (27), only modest severity agreement between echocardiography and CMR imaging in the assessment of primary MR severity (37 of 103 patients, 36%) was documented, with systematic overestimation by echocardiography. Notably, only CMR-derived RVol was shown to accurately predicted LV remodeling after MV surgery (27).

In ischemic MR, CMR imaging allows a comprehensive approach, as the presence of both significant ischemic MR (RF>35%) and myocardial infarct size (>30% of LV mass) is associated with a very high risk for all-cause mortality and/or heart transplant, despite MV surgery (28). On the other hand, patients with significant ischemic MR, but small myocardial infarct size (<15% LV mass) had a survival benefit with surgical MV intervention.

Importantly, in order to ensure accurate quantification of MR by CMR, it is necessary to bear in mind the importance of high-quality cine and phase-contrast modules and continuous quality control programs (29). Furthermore, caution should be taken in order to recognize and eliminate potential sources of error leading to inaccuracies in flow quantification associated with baseline phase offset errors, arrhythmias, VENC setting, high velocity jets in the ascending aorta, or improper contour segmentation (30).

Furthermore, CMR allows for the non-invasive quantification of structural remodeling of the myocardium with an expansion of the extracellular volume (ECV) fraction as a histopathologically validated surrogate for diffuse interstitial fibrosis (31). Incorporating ECV with quantitative MR severity assessment by CMR may aid in further risk stratification for adverse events prediction even in patients with secondary MR (32, 33).

Sometimes, symptoms appear disproportionate to resting MR severity, seemingly only mild or mild-to-moderate. Given the dynamic nature of secondary MR, evaluation of regurgitation should be carried out under optimized loading conditions, and it is usually recommended that invasive procedures should be deferred until any medical therapy has been optimized (34). In this setting, exercise stress echocardiography may reveal exercise-induced severe MR and pulmonary hypertension undetected at rest (35). An increase in MR severity (EROA >13 mm^2^) and dynamic pulmonary hypertension (systolic pulmonary artery pressure rise >60 mmHg) are predictors of poor outcome, regardless of the resting severity of secondary MR (36, 37).

## Therapeutic Considerations

Persistence or worsening of secondary MR despite OMT is the most important independent predictor of mid-term adverse outcomes. Conversely, early (<6 months) improvement is associated with increased survival, comparable with non-severe MR at baseline (38).

All therapeutic options should be considered to interrupt the vicious cycle of progressive LV volume overload, dilatation, and subsequent worsening MR. Beta-blockers, angiotensin-converting-enzyme inhibitors, and angiotensin receptor blockers can have an impact on secondary MR by reversing LV remodeling and are the only treatment with a Class I recommendation (39). Treatment with angiotensin receptor-neprilysin inhibitors holds promise, being associated with a reduction of secondary MR and LV end-diastolic volume index (40).

Cardiac resynchronization therapy (CRT) is aimed at restoring global and local LV synchronization and increasing global LV contraction efficiency. It has been shown that CRT can effectively reduce the severity of secondary MR among patients in NYHA class II-IV despite OMT, who remain in sinus rhythm where they are found to have LVEF ≤35%, QRS duration ≥130 ms and left bundle branch block; unfortunately, secondary MR persists in nearly 20–25% CRT recipients, and may even worsen in an additional 10–15% (41).

The benefits of surgery for secondary MR are controversial. As reverse remodeling probability correlates with LV size and duration of heart failure, early MV intervention has been proposed (42).

However, it has not been shown to modify the natural course of the disease. MV repair is associated with lower perioperative mortality, but a higher risk of recurrence. In contrast, replacement provides a more effective and sustained reduction of MR, although associated with higher long-term mortality (43). In the absence of large-scale studies directly comparing the two strategies, a meta-analysis including 1,730 patients with ischemic MR - the majority undergoing revascularization surgery – showed that MV replacement was associated with 35% higher mortality compared with the repair group (44).

In patients with multivessel coronary artery disease, complete coronary revascularization confers a clear survival benefit (45), and LV function improvement in ischemic cardiomyopathy should theoretically translate into a reduction of MR severity; nevertheless, compelling evidence supporting this hypothesis is still lacking.

As highlighted in the Euro Heart Survey (46), about 50% of patients with severe symptomatic MR are not referred to cardiac surgery, and leading causes are reduced LVEF, older age, and comorbidity. Current guidelines differ in terms of recommendations for surgery on secondary MR. ESC guidelines (47) recommend MV surgery only with concomitant bypass grafting as class I for patients with LVEF >30%, class IIa for those with LVEF <30% and evidence of myocardial viability, although in both cases based on expert consensus, with a “C” level of evidence, giving a preference for MV repair over replacement. AHA/ACC guidelines recommend MV surgery in class IIa for patients with secondary MR undergoing concomitant bypass or aortic valve surgery and in class IIb for patients with persistent symptoms (NYHA class III-IV) despite OMT, considering it reasonable to choose chordal-sparing MV replacement over repair (12).

## Percutaneous Edge-to-Edge Mitral Valve Repair

Over the last decade, percutaneous mitral valve repair (PMVR) has emerged as a viable treatment option for patients with secondary MR, and of many technologies, MitraClip™ (Abbott Vascular, Santa Clara, California) is currently the most widely used device. Using a transvenous, transseptal approach, one or more clips are used to approximate the free edges of the anterior and posterior leaflets, percutaneously replicating the Alfieri edge-to-edge surgical mitral repair technique (48).

In the EVEREST (Endovascular Valve Edge-to-Edge Repair Study) II trial, PMVR has been shown to be safer but less effective than surgical MV repair in a patient population with prevalent primary MR. However, in the smaller subgroup of subjects with secondary MR, PMVR, and surgery were comparably safe and effective (49). In the EVEREST II “high risk” study, symptomatic patients with severe MR at high risk for surgery and undergoing MitraClip showed a mortality rate lower than predicted for surgical treatment, an improvement in clinical symptoms, and significant LV remodeling (50). Several observational studies confirmed that MitraClip is associated with a high percentage of procedural success, improvement of hemodynamic and functional status in patients with severe secondary MR and high surgical risk (51, 52). In patients unresponsive to both OMT and CRT, MitraClip may induce a clinical benefit, with a reduction of natriuretic peptides and pulmonary artery pressure, improvement of NYHA functional class, and reverse LV remodeling (53). However, in the PERMIT-CARE (Percutaneous Mitral Valve Repair in Cardiac Resynchronization Therapy) (54), the lack of translation of functional outcome on mortality benefit among CRT non-responders reiterates the unmet need for more precise timing and a tailored patient selection for PMVR among. HF patients who are candidates for or have previously implanted CRT (55).

EVEREST II was mostly focused on primary MR and applied rigorous echocardiographic enrollment criteria aimed to ascertain adequate tissue leaflet quality, length, and mobility for “optimal” leaflet grasping (56). More recently, MitraClip has been performed mostly in secondary MR using expanded echocardiographic features falling outside the EVEREST I and II criteria (Table 1), yet demonstrating the potential for similar rates of safety and efficacy compared with EVEREST trials candidates (57).

**Table 1:** Determinants of percutaneous mitral valve repair efficacy in secondary mitral regurgitation.

**Optimal**	**Conditionally suitable**	**Unsuitable**
- NYHA II-III - Severe MR: EROA ≥ 0.4 cm RVol ≥ 60 ml; RF ≥ 50% - Non-ischemic cardiomyopathy - MV area >4 cm^2^ - Disproportionate MR - EROA/LVEDV ratio ≥0.14 - LVEDV index <96 ml/m^2^ - Central MR - Coaptation depth <11 mm - Coaptation length ≥2 mm - Mobile length of PL ≥10 mm - Preserved RV function - No PH - MIS <15 % - ECV <30%	- Exercise-induced severe MR and PH - Ischemic cardiomyopathy - MV area 3–4 cm^2^ - RV dysfunction with contractile reserve - Proportionate MR - Reversible PH - Eccentric MR - Coaptation depth <11 mm - Tenting area >1 cm^2^ - Mobile length of PL 6-10 mm - ECV >30% - MIS <30%	- NYHA IV, frequent hospitalizations for HF - VO2 peak ≤ 10 ml/kg/min - NT-proBNP >10.000 pg/ml - MV area <3 cm^2^ - Disproportionate LV disease - EROA/LVEDV ratio ≤ 0.12 - Mobile length of PL <6 mm - Coaptation depth ≥ 11 mm - Tenting area >2 cm^2^ - RV dysfunction without contractile reserve - Irreversible precapillary PH - MIS >30%

*ECV, extracellular volume; EROA, effective regurgitant orifice area; HF, heart failure; LVEDD, left ventricular end-diastolic diameter; LVEDV, left ventricular end-diastolic volume; MIS, myocardial infarct size; MV, mitral valve; MR: mitral regurgitation; NYHA, New York Heart Association; NT-proBNP, N-terminal pro-brain natriuretic peptide; PH, pulmonary hypertension; PL, posterior leaflet; RF, regurgitant fraction; RV, right ventricle; RVol, regurgitant volume; VO2, oxygen consumption*.

**Figure 1:**
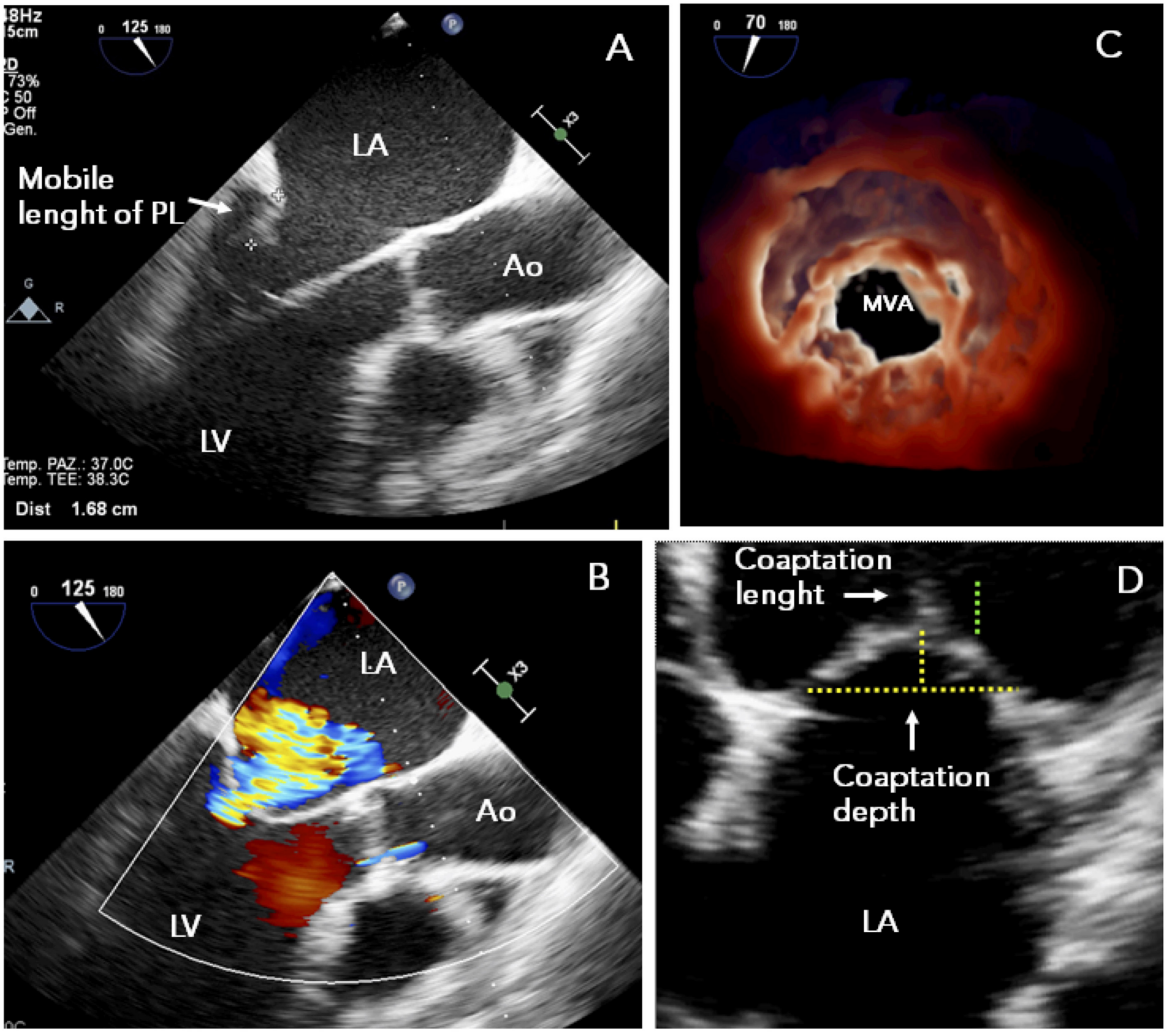
TTE study in a patient presenting with functional MR and mitral valve morphology “suitable” to MitraClip: **(A)** posterior leaflet mobile length ≥10 mm; **(B)** symmetrical tethering of non-calcified leaflets with central regurgitant jet; **(C)** adequate MVA (>4 cm^2^); **(D)** preserved leaflet coaptation. Ao, ascending aorta; LA, left atrium; LV, left ventricle; MR, mitral regurgitation; MVA, mitral valve area; PL, posterior leaflet; TEE, transesophageal echocardiography.

**Figure 2:**
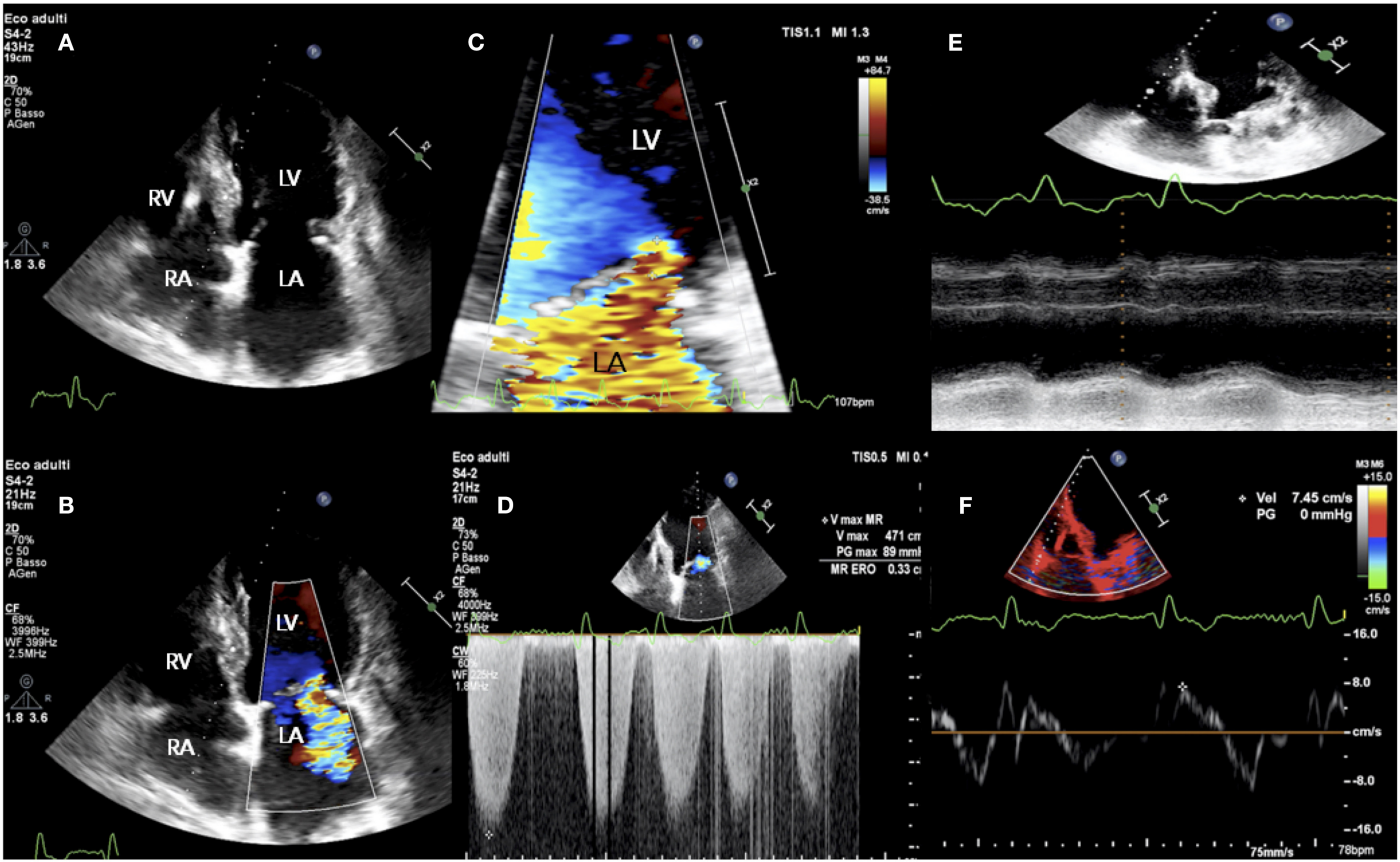
TTE study in a patient presenting with acute signs and symptoms of HF, NYHA class III, NT-proBNP 6,459 pg/ml, and “disproportionate” functional MR. **(A)** LV end-diastolic volume of 175 ml; **(B–D)** holosystolic, central jet of MR and EROA of 0.34 cm^2^; **(E,F)** RV longitudinal dysfunction with TAPSE 10 mm and S' 7 cm/sec. EROA, effective regurgitant orifice area; HF, heart failure; NYHA, New York Heart Association; NTproBNP, N-Terminal pro-B-type natriuretic peptide; TAPSE, tricuspid annular plane systolic excursion; TR, tricuspid regurgitation; TRV, TR velocity; TTE, transthoracic echocardiography; other abbreviations as in previous figures.

ESC guidelines for the management of HF and valvular heart disease both gave PMVR a class IIb recommendation (level of evidence C) (39, 47) placing MitraClip at the same level as surgery in patients with secondary MR and reduced LVEF who have a “suitable” valve morphology by echocardiography (Figures 1, 2).

More recently, long-awaited results from the two recent randomized controlled trials MITRA-FR (Percutaneous Repair with the MitraClip Device for Severe Functional/Secondary Mitral Regurgitation) and COAPT (Cardiovascular Outcomes Assessment of the MitraClip Percutaneous Therapy for Heart Failure Patients with Functional Mitral Regurgitation) (23, 58), directly comparing PMVR+OMT vs. OMT in patients with chronic HF, systolic LV dysfunction and secondary MR, reported controversial findings. The MITRA-FR showed no difference in the primary endpoint, which was a composite of 1-year re-hospitalization and all-cause death. Compared to the OMT group, COAPT showed a significant reduction in 2-year HF hospitalization in the PMVR+OMT group and in all-cause mortality.

Nishimura and Bonow (59) commented that, upon enrollment, a significantly higher percentage of patients were truly refractory to OMT in the COAPT than in MITRA-FR and likely more responsive to PMVR. Moreover, despite similar acute procedural success (achieved in >90% of cases by using ≤2 clips), residual MR of grade 3+ or higher at 12 months was significantly more frequent in MITRA-FR than in COAPT (17 vs. 5%). Indeed, a residual 2+ MR 1-year after MitraClip implantation is known to be associated with a worse outcome (60). In both trials, little information was provided by the authors on pre- and post-procedural right ventricular (RV) function and on residual MV pressure gradient. Overall, the most compelling explanation for alleged controversial findings of the two randomized trials seems to be related to the differences in MR grade and LV size at enrollment.

**Figure 3:**
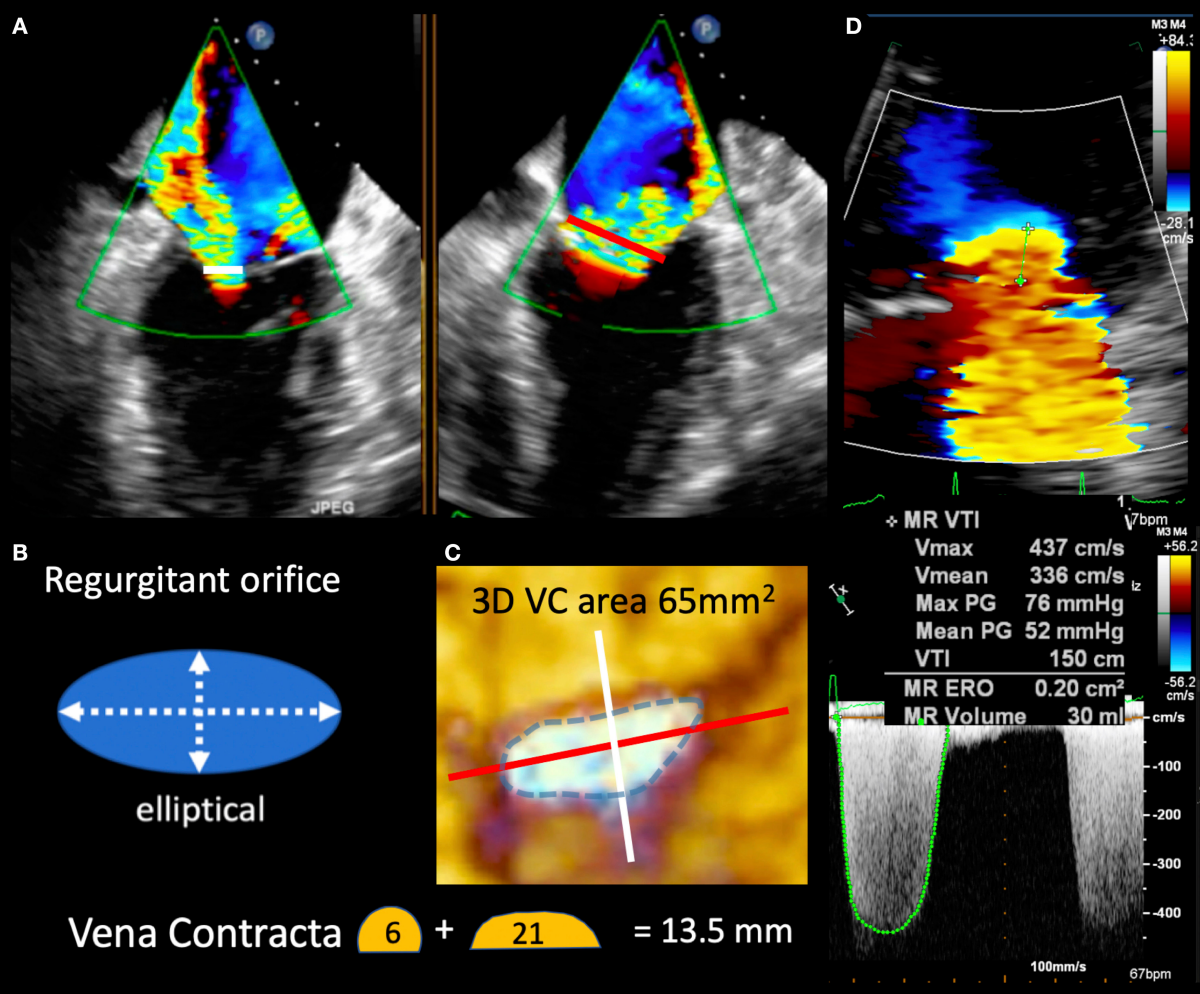
TEE image in “disproportionate” secondary MR demonstrating the vena contracta in two planes (white and red lines) seen to be longer in the commissural view. **(B,C)** Depict the regurgitant orifice morphology as elliptical and explain the vena contract size differences seen in **(A)**. 3D vena contracta area of 65 mm^2^ better reflects the large elliptic regurgitant orifice compared with EROA of 20 mm^2^ by standard 2D Doppler method seen in **(D)**.

**Figure 4:**
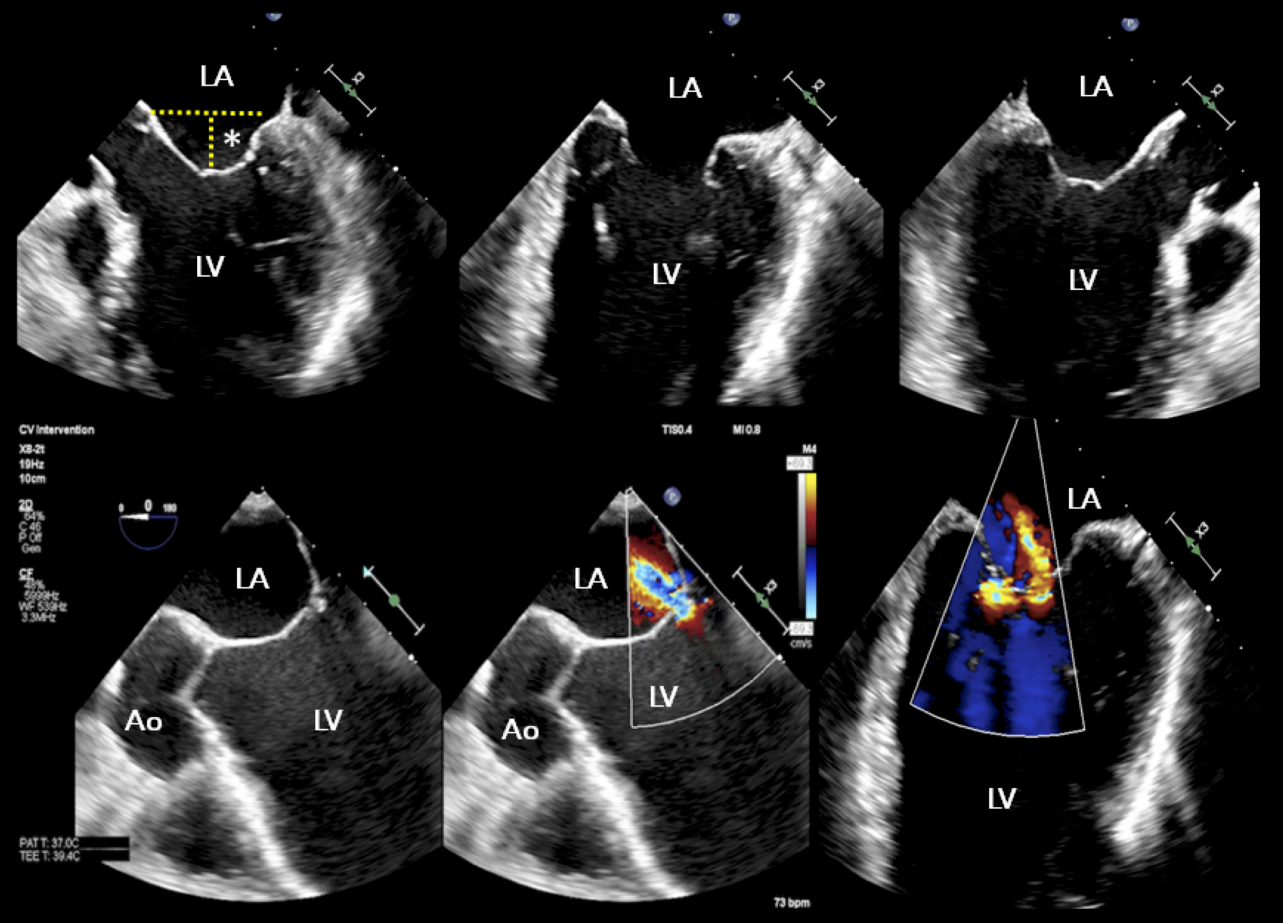
TEE study of a patient with advanced heart failure and reduced ejection fraction, NYHA IV, NT proBNP 23,000 pg/ml, and functional “proportionate” MR. The LV appears severely dilated with coaptation depth >15 mm. A conservative management approach was recommended. Asterisk indicates coaptation depth; other abbreviations as in previous figures.

**Figure 5:**
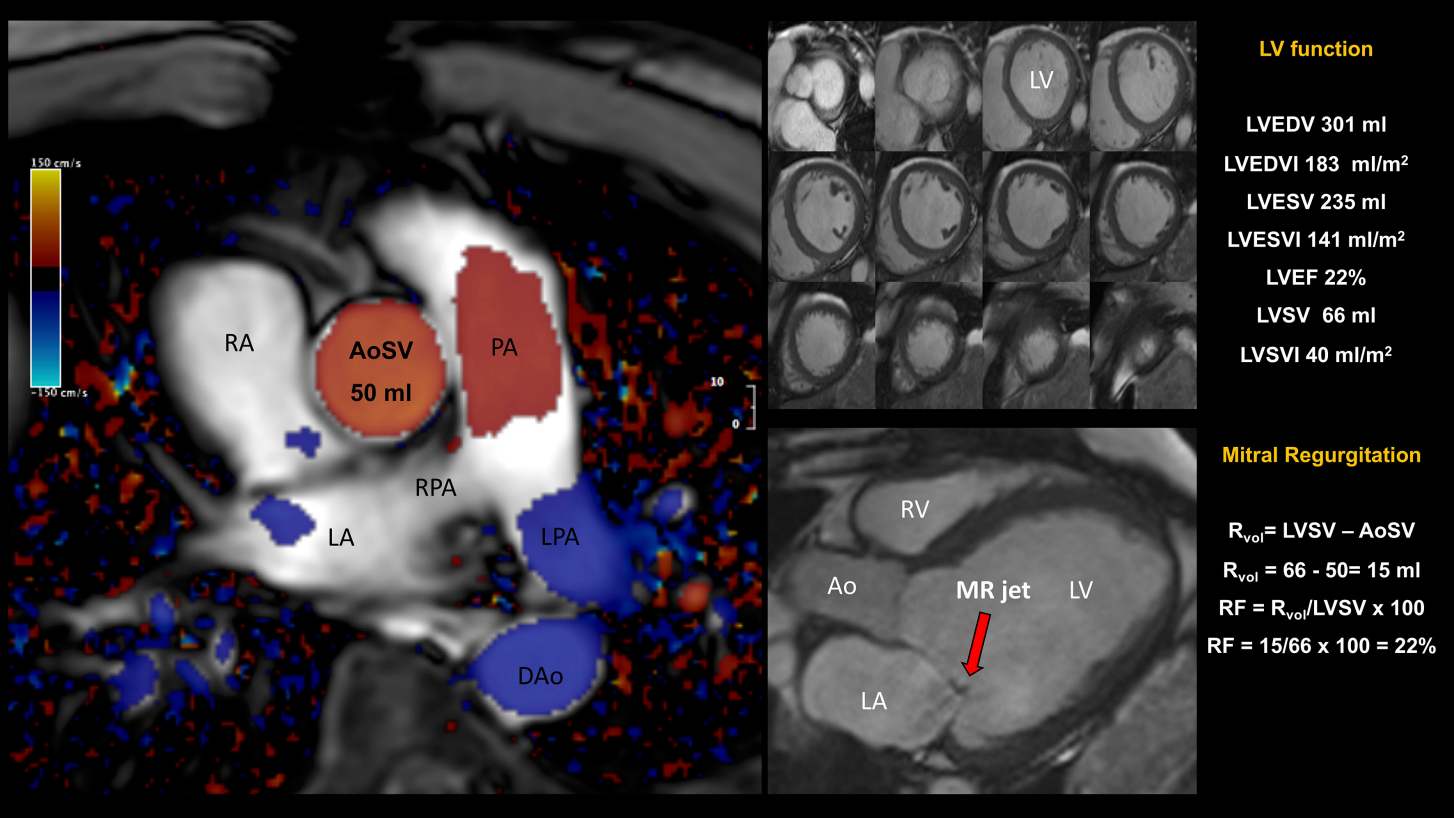
Patient admitted for NSTEMI with severe eccentric MR by TTE. CMR showed (i) a severely dilated left ventricle (LVEDVI 183 ml/m^2^) and global impaired systolic function (LVEF 22%, LVSV 66 ml); (ii) a dilated left atrium; (iii) asymmetric tethering of the posterior leaflet and eccentric posteriorly directed MR. Quantification of mitral RVol was performed using the so-called indirect approach, comparing LVSV (derived by planimetry of short-axis cine) to forward flow (derived by phase-contrast CMR) across the aortic valve (aortic stroke volume, AoSV). This is the preferred method for MR quantitation as generally not affected by the presence of concomitant valvular regurgitant lesions. The total volume of blood ejected from the LV, i.e., LVSV, was computed as the difference between LVEDV and LVESV. In this example, LVSV was 66 ml. CMR phase contrast imaging yielded an AoSV of 50 ml, mitral RVol of 16 ml, and a RF of 24%. The mitral RVol was computed as the difference between the LVSV and AoSV and, in this example, was 70 mL. In addition, RF was calculated using the RVol and the LVSV; in this example, RF was 24%. Overall, CMR findings were in keeping with dilated cardiomyopathy and mild secondary MR. AoSV, aortic stroke volume; CMR, cardiovascular magnetic resonance; DAo, descending aorta; LGE, late gadolinium enhancement; LPA, left pulmonary artery; LVEDV, left ventricular end-diastolic volume; LVEDVI, left ventricular end-diastolic volume index; LVEF, left ventricular ejection fraction; LVESV, left ventricular end-systolic volume; LVESVI, left ventricular end-systolic volume index; LVSV, left ventricular stroke volume; LVSVI, left ventricular stroke volume index; NSTEMI, Non-ST Elevation Myocardial Infarction; PA, pulmonary artery; RF, regurgitant fraction; RPA, right pulmonary artery; RVol, regurgitant volume; other abbreviations as in previous figures.

Grayburn (61) delineated two major profiles of significant secondary MR:

a) “disproportionate” MR refers to a regurgitant volume non-commensurate to the amount of LV dilatation. This group does include patients exhibiting regurgitant volumes disproportionately higher than the degree of LV dilatation. Such patients are likely to mainly benefit from a therapy targeted to the mitral valve with PMVR, beyond OMT and/or CRT (Figures 2, 3);

b) “proportionate” MR refers to a regurgitant volume totally commensurate to LV enlargement. This group would likely benefit the most from strategies aimed at reducing LV size (i.e., OMT and CRT) alone, not directed to MV apparatus (Figures 4, 5).

From this viewpoint, most patients enrolled in MITRA-FR would have proportionate MR, where mean LVEDV and EROA (252 ml and 31 mm^2^, respectively), reflected MR commensurate to the degree of LV dysfunction and likely representing advanced ventricular disease. Conversely, subjects included in COAPT trial had LVEDV 30% smaller (mean 192 ml) and EROA 30% larger (mean 41 mm^2^) than MITRA-FR patients, and MR appeared to be far “out of context” to the degree of LV disease.

Two later meta-analyses (62, 63), documented a significant reduction in mortality and risk of HF hospitalization among patients who received MitraClip+OMT as compared with OMT in secondary MR. The prognostic efficacy of PMVR seems to be a function of time, with mortality rates continuously diverging over a longer follow-up period (62). Consistent with Grayburn's conceptual framework (61), patients with smaller LV end-diastolic volume index achieved the most considerable reduction in mortality, whereas larger baseline LV size undermined the expected benefit of PMVR (63) (Figure 4). Importantly, baseline indexed LVEDV was found to explain most of the between-study heterogeneity. Such findings were paralleled by the large GIOTTO (Italian Society of Interventional Cardiology (GIse) Registry Of Transcatheter Treatment of Mitral Valve RegurgitaTiOn) registry (64), where indexed LVEDV was an independent predictor of adverse 30-day outcome, together with EuroSCORE II and prolonged stay in the intensive care unit.

## Beyond Baseline Evaluation of MV and LV Morphology

Patients with HF and secondary MR exhibit a higher-risk profile, having significant comorbid conditions such as renal or pulmonary disease (65). The sole assessment of baseline MV anatomy and LV morphology does not accurately predict the outcome before and/or after MitraClip deployment.

### Variables Before PMVR

Although patients with severe LV dysfunction (LVEF <20%) can exhibit both clinical and hemodynamic improvement after MitraClip (66), it has been extensively documented that older patients with more advanced HF, chronic kidney disease, NYHA functional class IV and severe tricuspid regurgitation have a dismal prognosis (67).

The presence and extent of replacement myocardial fibrosis, as assessed by late gadolinium enhancement at CMR, is more frequent in secondary than in primary MR and predicts an adverse outcome after PMVR (28, 68).

Among patients with end-stage advanced HF, pre-treatment with inotropes is associated with safer and more effective MitraClip deployment, reduction of HF hospitalizations, and improvement of symptoms after 6 months (69).

In a cohort of 414 patients with secondary MR, non-ischemic MR heralded greater functional benefit as compared with the ischemic etiology, where LVEF ≤ 25%, LVEDV by echo > 216 ml, NT-proBNP ≥ 10,000 pg/ml and atrial fibrillation were the strongest baseline variables associated with 2-year cardiovascular death (70).

The assessment of RV systolic function is highly relevant for PMVR-related outcome (Figure 2). Peak systolic velocity <9.5 cm/s by tissue doppler imaging was independently associated with cardiovascular mortality in a cohort of high surgical risk patients affected by severe secondary MR undergoing PMVR (71). Despite similar rates of procedural success (>90%), patients with RV dysfunction (TAPSE ≤ 16 mm) are less likely to derive a clinical benefit from Mitraclip in terms of functional capacity and survival than patients with normal RV function (TAPSE >16 mm) (72). Conversely, in patients with dilated cardiomyopathy and biventricular dysfunction, the presence of RV contractile reserve under dobutamine stress (20 μg/kg/min) offers a significantly better prognosis (73). Pre-procedural cardiopulmonary exercise test may be useful in the identification of the optimal candidate to Mitraclip, and a peak VO_2_ value of 10 ml/kg/min was identified as the best cut-off for prediction of cardiac and all-cause death and HF hospitalization (74).

Stress echocardiography, beyond the definition of MR relevance, may also help to identify patients who derive benefit from MitraClip: subjects who experience a reduction in MR severity during preprocedural low-dose stress (dobutamine or handgrip) echocardiography remain more symptomatic after intervention compared with those presenting with stable or increased MR during preprocedural stress, likely because in these patients MR might have contributed less to their symptoms during exercise, thus explaining the lack of benefit from a technically successful MitraClip procedure (75). Similarly, stress echocardiography may identify the optimal timing of PMVR at an earlier stage: patients with moderate resting developing severe exercise-induced MR during handgrip echocardiography have an improved treatment response after MitraClip (76).

### Variables Undergoing Modification After PMVR

Immediately after MitraClip implantation, acute post-procedural LV dysfunction may occur and is more likely in patients with advanced HF. However, this acute hemodynamic deterioration - known as afterload mismatch - is transient and does not imply any difference in perioperative and long-term outcomes (77).

Reverse LV remodeling after MitraClip procedure occurs in ~50% of patients and is associated with far lower mortality (27%) than adverse remodeling (67%, *p* < 0.001) at a median 32-month follow-up (78). In a large multicenter registry, reverse LV remodeling was documented in 43% of patients with secondary MR and associated with a lower 2-year all-cause mortality (*P* = 0.039) and risk of re-hospitalizations (*P* = 0.02) compared with patients with no remodeling (79). In the same registry, the female gender (*P* = 0.004), non-ischemic etiology of MR (*P* = 0.007), and LV end-diastolic diameter <75 mm (*P* = 0.044) were all independent predictors of reverse LV remodeling (79). Consistent findings were documented in a recent cohort of patients with both primary and secondary MR (80), as recurrent/residual MR after 12 months (*P* = 0.01), male gender (*p* = 0.050) and LVEF <20% (*P* = 0.046) were independent predictors of absence of LV reverse remodeling; in the subgroup of patients with secondary MR, only residual severe tricuspid regurgitation inversely predicted LV reverse remodeling.

TAPSE monitoring after MitraClip identified RV function decline in 20% and improvement in 55% of cases, and also changes in RV function after PMVR have been identified as independent predictors for survival (81). This further supports the role of preprocedural low-dose dobutamine stress (82, 83) as a potential gatekeeper test to reveal a subclinical reduction in RV contractile reserve and to inform on whether CRT, MitraClip or immediate left ventricular assist device (LVAD) therapy should be primarily indicated in cases of more advanced LV disease (84). Indeed, optimal patient selection and timely decision making are essential to prevent RV dysfunction which may not qualify for subsequent LVAD support (85), and avoid RV failure after LVAD implantation, that is associated with increased perioperative mortality, coagulopathy, altered drug metabolism, diuretic resistance, and poor quality of life (86). Nevertheless, the safety of the MitraClip system after LVAD implantation has been documented, with no need for additional mitral valve surgery (84, 87).

## Future Directions

Although all proven to be effective in reducing secondary MR, the efficacy of OMT, CRT, and PMVR is highly dependent on patient selection. The optimal timing of these therapeutic strategies has yet to be defined. OMT is appropriately the first-line approach. CRT is then recommended on the basis of clinical symptoms, LV systolic function, and electrocardiographic criteria. Finally, PMVR comes after the failure of the aforementioned treatments for LV dysfunction and dyssynchrony.

Future research should aim to identify a tailored approach, as the progression of the underlying disease may hamper the efficacy of MitraClip to interrupt the vicious cycle of progressive deterioration in LV function over time.

Two large-scale randomized studies will soon provide additional outcome data on PMVR with MitraClip: (1) the RESHAPE-HF2 (A Clinical Evaluation of the Safety and Effectiveness of the MitraClip System in the Treatment of Clinically Significant Functional Mitral Regurgitation - NCT01772108) trial, enrolling patients with clinically significant functional MR (moderate-to-severe or severe mitral regurgitation), as defined by European Association of Echocardiography, and different LVEF thresholds according to symptomatic status (NYHA Class II: LVEF 15–35%; NYHA Class III/IV: LVEF 15–45%) and excluding patients on the basis of the 6-min walk (when walking distance >475 m or unable to perform it); (2) the MATTERHORN trial (A Multicenter, Randomized, Controlled Study to Assess Mitral valve reconstruction for advanced Insufficiency of Functional or ischemic Origin - NCT02371512), comparing PMVR with surgery in patients at high surgical risk with LVEF ≥ 20% and clinically significant MR of primarily functional etiology.

In the meantime, newer PMVR devices are stepping through phases of clinical research. After Mitraclip, the two most widely used PMVR devices are annuloplasty systems: the Carillon Mitral Contour System® (Cardiac Dimensions Inc., Kirkland, WA, USA), deployed in the coronary sinus and the Cardioband® (Edwards Lifesciences, Irvine, CA, USA), implanted on the posterior annulus through a transeptal approach. Among 186 propensity-score matched patients with secondary MR, the use of Cardioband was associated with a reduction of 12-month mortality (OR 0.30, CI: 0.09–0.98, *p* = 0.032) and risk of HF hospitalization (OR 0.57, CI: 0.28–0.97, *p* = 0.03) compared with MitraClip, and differences were more evident in the subgroup of patients with LVEF≤30% (88). Given the different mechanisms of action of the two systems and the limited sample of the study population, results must be cautiously interpreted. More recently, another edge-to-edge PMVR device, the Pascal® (Edwards Lifesciences, Irvine, California) system, received CE marking. The PASCAL transcatheter valve repair system harnesses 2 clasps and paddles to achieve plication of the MV leaflets, while placing an anatomic spacer to fill the regurgitant orifice between the native valve leaflets (89). Notably, in the CLASP study enrolling a cohort of 109 patients with severe symptomatic MR (67% secondary MR), the PASCAL system yielded significant MR reduction - with 100% of patients achieving MR ≤2+ and 80% MR ≤1+ sustained at 1 year - and was associated with high survival, low complication rates and enduring improvements in functional status and quality of life at 1 year (90).

Overall, the selection of the appropriate patient and valvular anatomy for each device does represent the next challenge to be addressed by heart valve specialists and PMVR experts for a full mastery of MR management.

## The Selection of the Optimal Candidate

There is now substantial evidence that PMVR with MitraClip is highly effective in reducing secondary MR in patients with HF (91). However, the translation of such a favorable result in a lasting clinical benefit is far from being consistently predictable.

**Figure 6:**
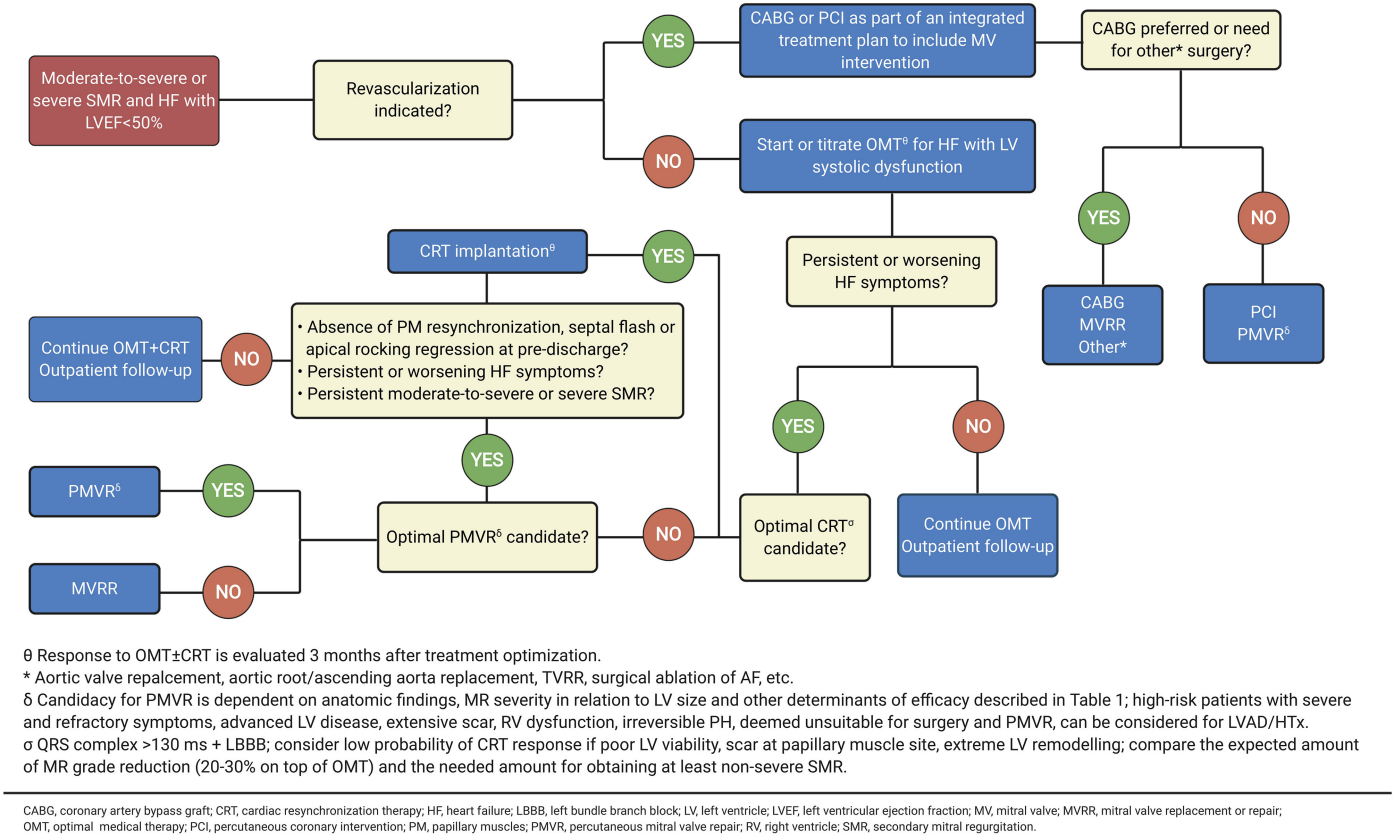
Intervention for symptomatic secondary MR. Adapted from 2020 focused update of the 2017 ACC expert consensus decision pathway on the management of mitral regurgitation (89) (Illustration created with https://biorender.com/).

Currently, in secondary MR, the optimal candidate to PMVR is a patient with chronic HF, LVEF <35%, severe MR, no residual ischemia or no further myocardial revascularization achievable, refractory symptoms despite OMT and CRT, and therefore deemed at high surgical risk but with anatomically suitable MV anatomy for MitraClip intervention (Figure 6, Supplementary Videos 1, 2). Patients at an earlier stage of the disease should be carefully monitored, and where stress echocardiography may be helpful to identify those more likely to progress and, therefore, to benefit from early interventional treatment.

**Figure 7:**
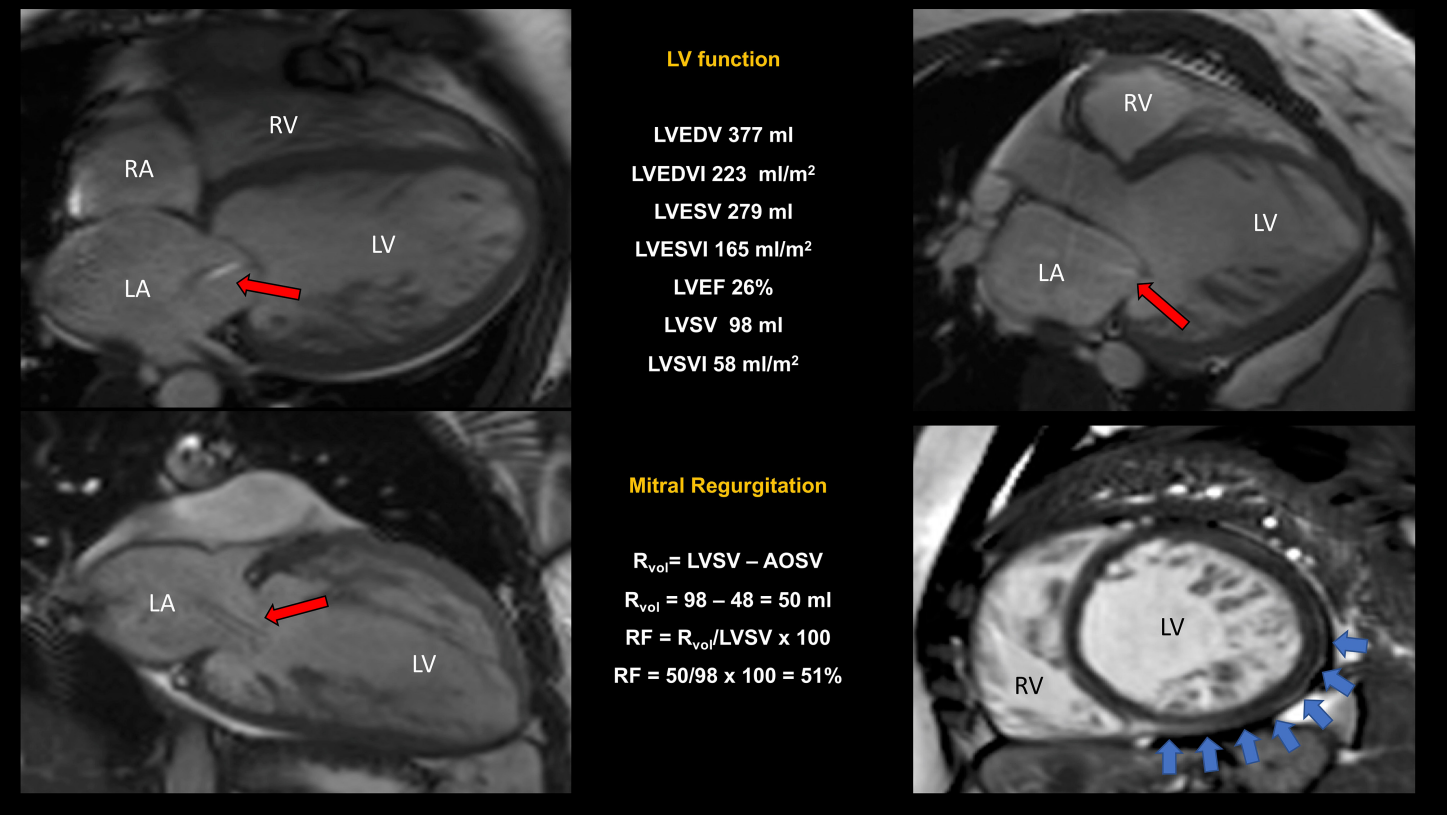
CMR study of non-compaction cardiomyopathy (NC/C ratio 4) with severely dilated and impaired LV and right ventricular involvement. Quantification of MR was performed using the indirect approach (see legend to Figure 5 for further explanation). In this example, we reported a “proportionate” severe (RF 51%) secondary MR (red arrows indicating the regurgitant jet). A dilated pulmonary artery (36 mm) was suggestive of pulmonary hypertension. Non-ischemic scar inferiorly and laterally (blue arrows). Conservative management was preferred. NC/C, non-compact/compact; other abbreviations as in previous figures.

**Figure 8:**
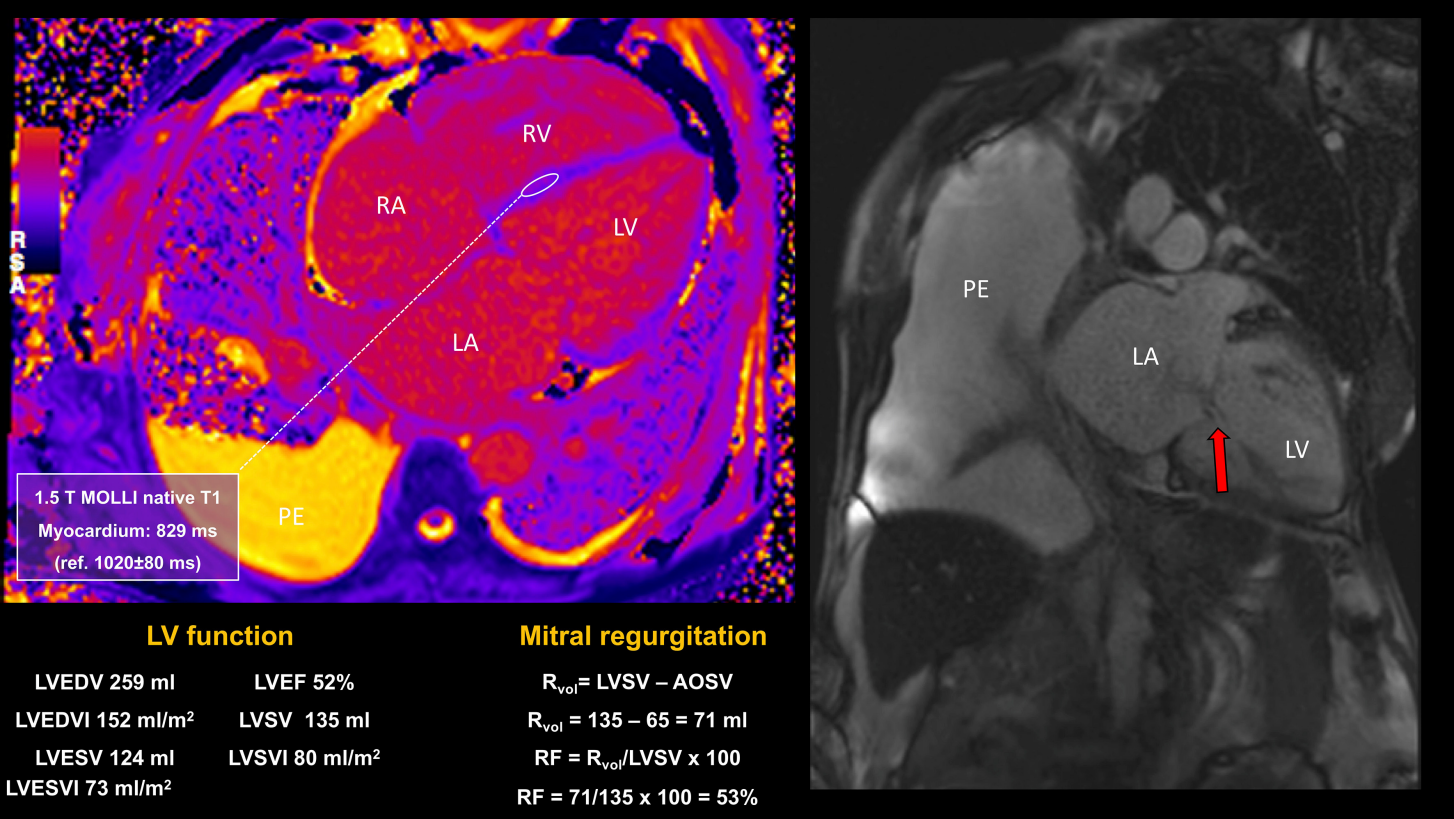
CMR study in a patient presenting with signs and symptoms of congestive heart failure, atrial fibrillation, severe MR, and with history of sickle cell disease and multiple blood transfusions. CMR showed bilateral pleural effusions, severe biatrial and biventricular dilatation, biventricular impairment (LVEF 52%; RVEF 39%) in the context of severe mitral regurgitation (RF 53%) and tricuspid regurgitation, early diffuse cardiac iron loading by T1 mapping and moderate hepatic iron loading. Despite successful MitraClip implantation, the progression of myocardial iron overload led to the worsening of symptoms and recurrent HF hospitalizations. PE, pleural effusion; RVEF, right ventricular ejection fraction.

**Figure 9:**
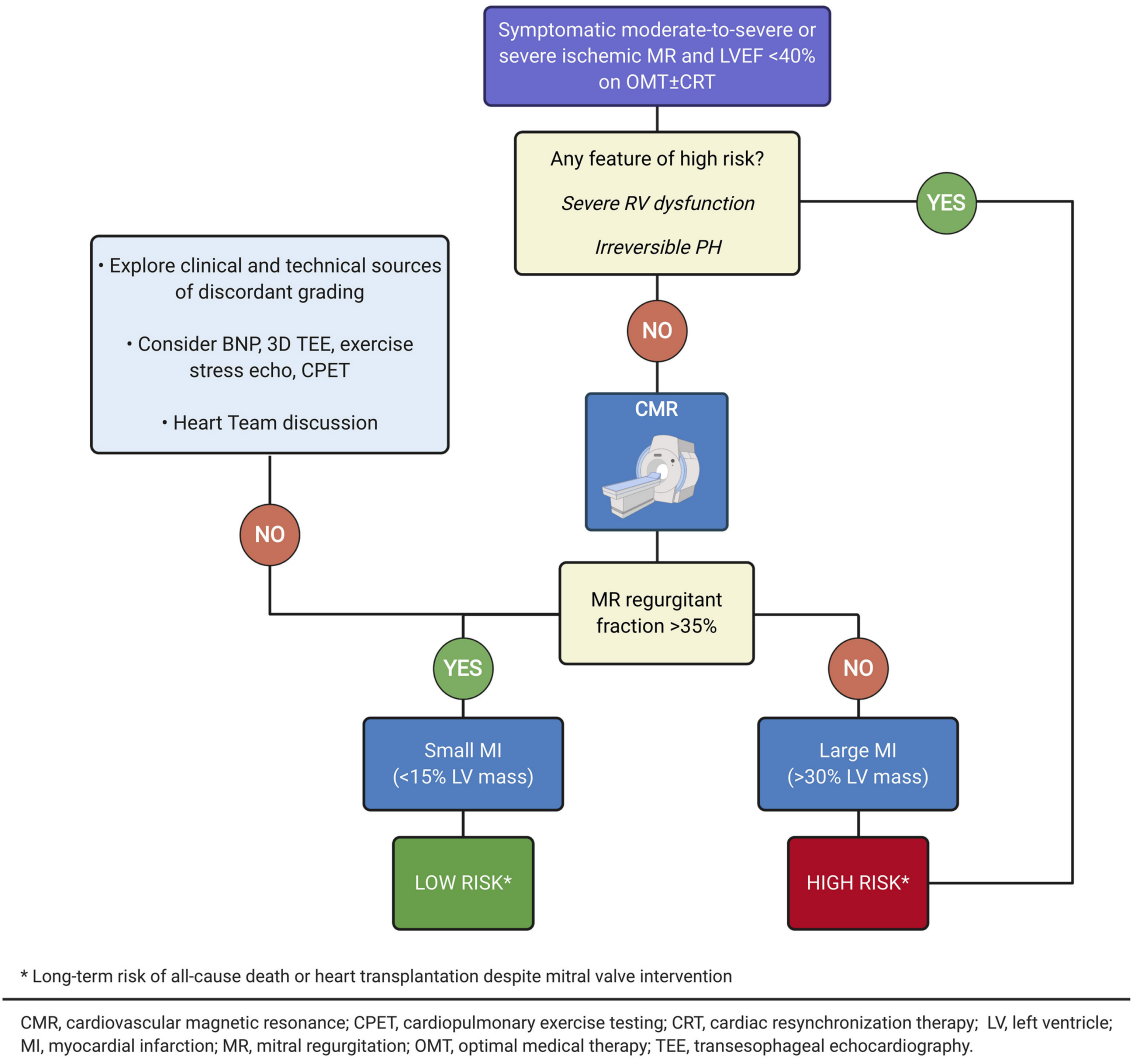
Risk stratification of patients with advanced ischemic cardiomyopathy by cardiovascular magnetic resonance imaging (Illustration created with https://biorender.com/).

Beyond anatomical criteria, clinical features may help to identify the optimal timing for PMVR and predict its efficacy (Table 1, Figures 7, 8). An accurate assessment of MR is essential. While EROA and RVol are the gold standards in MR severity assessment, the hemodynamic load (supported by RF assessment) and the extent and presence of advanced LV disease have recently been emphasized in recent randomized trials as key factors in determining the benefits of targeting the LV alone (OMT + CRT) or additionally the mitral valve (PMVR). However, clinical experience and published data also highlight the need to consider several additional factors, including the presence of severe RV dysfunction, advanced LV impairment (typically associated with very high BNP values), and an irreversible precapillary component of pulmonary hypertension (92), that can attenuate the benefit of PMVR and predict potential futility. Where doubt remains, functional testing to assess ventricular reserve (75, 83) and dynamic RV-to-pulmonary circulation uncoupling (82, 93) can be useful for further risk stratification. Furthermore, risk associated with ischemic MR has been more comprehensively described as an interaction between MR severity and myocardial infarct size by CMR imaging, lending an opportunity for improved risk stratification beyond LV volumes and clinical parameters, and for the individualization of treatment decision (Figure 9).

Hence, clinical, functional, and multi-modality imaging assessment of the patient should be part of a comprehensive and meticulous workup to help direct the clinicians to those patients who could derive the maximal benefit from PMVR.

Nowadays, PMVR indications are expanding, and MitraClip implantation might be considered immediately after worsening symptoms despite OMT in order to avoid the progression toward refractory HF. Further evidence from adequately designed and powered randomized trials is warranted to confirm the efficacy of PMVR for the treatment of secondary MR and to provide more detailed information indications about optimal patient selection and appropriate timing of intervention.

## Author Contributions

Conception and design: FR, TS, MZ, and SG. Review of the literature and interpretation of data: FR, LC, MK, GD, CF, and BR. Drafting of the manuscript: FR, TS, MZ, MA, SG, and BR. Critical manuscript revision for important intellectual content and approved the final version of the manuscript by all authors.

## Conflict of Interest

The authors declare that the research was conducted in the absence of any commercial or financial relationships that could be construed as a potential conflict of interest.
